# Gay Community Connectedness, Internalized Homonegativity, and HIV Pre-Exposure Prophylaxis (PrEP) Attitudes and Use Among Men Who Have Sex with Men in Georgia: A Mediation Analysis

**DOI:** 10.1007/s10461-025-04870-0

**Published:** 2025-09-12

**Authors:** Mohammad Rifat Haider, Golam Sarwar, Liyuan Wang, Md. Sharful Islam Khan, Monique J. Brown, Nathan Hansen, Jeremy J. Gibbs

**Affiliations:** 1 Department of Health Policy and Management, College of Public Health, University of Georgia, Athens, GA USA; 2 Department of Health Promotion and Behavior, College of Public Health, University of Georgia, Athens, GA USA; 3 Program for HIV/AIDS, Health Systems and Population Studies Division, International Centre for Diarrhoeal Diseases Research, Dhaka, Bangladesh; 4 Department of Epidemiology and Biostatistics, Arnold School of Public Health, University of South Carolina, Columbia, SC USA; 5 School of Social Work, University of Georgia, Athens, GA USA; 6 Department of Health Policy and Management College of Public Health, University of Georgia, 100 Foster Rd Wright Hall 301B, Athens, GA 30602 USA

**Keywords:** Gay community connectedness, Attitudes towards PrEP, PrEP use, Internalized homonegativity, Men who have sex with men

## Abstract

Georgia has one of the highest HIV diagnosis rates in the U.S., yet only 33% of Pre-Exposure Prophylaxis (PrEP) users in the Deep South live there. This study aims to determine the potential mediational role of internalized homonegativity and attitudes towards PrEP between gay community connectedness and PrEP use among men who have sex with men (MSM) living in Georgia. Participants (*N* = 121) completed an online survey in June 2020. PrEP use (yes/no) was defined by the question “the last time you had sex, were you taking PrEP, like Truvada?” The study also used the “8-item Identification and Involvement with the Gay Community scale”, “9-item internalized homophobia scale”, and a “3-item PrEP attitude scale”. Path analysis was performed using Stata 17.0. After adjusting for sociodemographic characteristics, positive associations were observed between attitudes towards PrEP and PrEP use (β = 0.11; p = < 0.001), and gay community connectedness (GCC) and PrEP attitudes (β = 0.17, *p* < 0.001). Conversely, negative associations were found between GCC and internalized homonegativity (β=−0.71, *p* < 0.001), and internalized homonegativity and PrEP use (β=−.01, *p* = 0.031). GCC was not significantly associated with PrEP use (β=−.01; *p* = 0.196). However, the indirect effects of GCC on PrEP use through PrEP attitudes (β = 0.01, *p* < 0.001) and internalized homonegativity (β = 0.02, *p* = 0.041) were statistically significant. These findings indicate that PrEP attitudes and internalized homonegativity mediate the relationship between GCC and PrEP use among MSM. Thus, improving attitudes toward PrEP and reducing internalized homonegativity through fostering GCC among MSM may improve PrEP use and persistence.

**Supplementary Information:**

The online version contains supplementary material available at 10.1007/s10461-025-04870-0.

## Introduction

Human Immunodeficiency Virus (HIV) remains a pressing public health problem in the United States (U.S.) and disproportionately affects certain population groups due to several social and structural issues, including HIV stigma, discrimination, poverty, and limited access to preventive health services [1]. Although men who have sex with men (MSM) account for only 2% of the total U.S. population, they constitute 67% of the estimated new HIV cases and 83% of the prevalent HIV cases among U.S. men in 2022 [1, 2]. Georgia, a “Deep South” U.S. state, had the second highest HIV diagnosis rate (27.4%) among the states in 2022 [3]. Despite being 2.5% of the total population in Georgia, MSM accounted for 66% of the total HIV diagnoses in 2022 [4].

Inadequate or lack of access to bio-behavioral prevention or harm reduction strategies, including pre-exposure prophylaxis (PrEP), HIV/sexually transmitted infection (STI) testing, and harm reduction services, contributes to observed disparities in HIV rates. Behavioral harm reduction services dedicated to addressing HIV alone have not been enough to control HIV cases due to behaviors of key populations at risk of HIV, such as engaging in condomless sex and sexualized drug use (SDU) [5–7]. SDU denotes the usage of drugs before and during planned sex to initiate sexual activities and to enhance and prolong sexual pleasure [8]. SDU is associated with increased HIV transmission risk through condomless sex [9–11]. Therefore, adoption of the bio-behavioral prevention approaches (PrEP, HIV/STI testing) and harm reduction services specifically focused on prevention, risk reduction, and health promotion could reduce the harm associated with drug use and risky sexual behaviors, and interrupt HIV transmission risk without requiring abstinence or reduction of drug use and sexual behaviors [12–14].

Hence, an emphasis has been given globally on the implementation of PrEP, particularly among MSM. PrEP is an evidence-based biomedical prevention approach to prevent acquiring HIV through sexual exposure [15]. The efficacy of oral PrEP for MSM has already been proven in several studies and clinical trials (i.e., IPERGAY, PROUD), demonstrating an 86% reduction of HIV incidence among MSM [16–20]. Considering further evidence of the effectiveness and acceptability of PrEP, the World Health Organization (WHO) recommended PrEP for all populations disproportionately impacted by HIV [21]. Despite the high HIV burden, the PrEP usage rate in Georgia was 203/100,000 population with a PrEP-to-Need Ratio (PNRs) of 7.5, demonstrating a very high unmet need [22].

Diverse factors related to PrEP uptake and persistence have been identified, including factors that promote PrEP use, such as HIV risk perception and PrEP knowledge and awareness. However, some factors hinder PrEP use, such as stigma related to HIV and PrEP, discrimination at health facilities, lack of support from family and society, inadequate access to PrEP services, high costs of PrEP medicines, lack of health insurance, and concerns about anticipated adverse effects and long-term consequences of PrEP medicines [23, 24]. Furthermore, social networks were also found to influence PrEP awareness and acceptability among underserved populations. Research among MSM has revealed the key role of social networks in providing PrEP information and support for MSM [25–27]. “Gay community connectedness” (GCC) denotes a “sense of in-group status or belongingness to a broader gay community, an affiliative subgroup of the community, or other sexual minorities” [28, 29]. “Gay community connectedness” is considered a protective factor for the health and well-being of MSM as well as a fundamental resource for coping with societal challenges, access to essential health information, and psychological support [28, 30, 31]. Higher gay community connectedness and attachment were linked to increased social support and resilience, and reduced internalized homonegativity, depression, and stigma [30]. A significant positive association was revealed between gay community connectedness and PrEP uptake [32]. Gay individuals with high community attachments and connectedness could have gained increased access to reliable PrEP information and resources [33, 34]. Another study showed that the intersection between the gay and ethnic communities can influence PrEP use among Latinx MSM. This suggests that the role of the community can be modified based on different cultural factors and situations [32]. A qualitative study conducted among U.S. Latino gay and bisexual men revealed that those who used PrEP felt more connected to the community [35]. Moreover, the engagement of peer health providers has demonstrated effectiveness in enhancing willingness to accept PrEP and increasing PrEP uptake, as observed in a qualitative study conducted in the U.S., which demonstrated that peer-driven interventions, led by trained peers who are well-informed about PrEP, could improve PrEP uptake among MSM. This approach could address health disparities and potentially decrease HIV transmission in Black/African American and Hispanic/Latino MSM communities [36]. However, community and social networks can increase the stigma surrounding PrEP by relating it to promiscuity. This can result in gossip and rumors that intensify feelings of shame, making it more difficult for individuals to access and use PrEP [26, 37]. Additionally, medical/health misinformation may spread across the community through social media and online retail sites, leading to myths and medical mistrust [38–40], thus reducing the willingness to use PrEP.

Internalized homonegativity refers to “the gay person’s direction of negative social attitudes toward the self, leading to a devaluation of the self and resultant internal conflicts and poor self-regard” [41]. Several factors were suggested as predictors of internalized homonegativity, including low identity resilience, perceived negative social representation, outness, lack of social support, and discrimination [42]. Research has demonstrated adverse social, psychological, and sexual health consequences, including sexual risk behavior, substance use, compromised mental health status, and relationship problems due to internalized homonegativity [43–48]. Several studies have reported an association between internalized homonegativity and reduced PrEP uptake [49–52], as well as reduced PrEP awareness [53, 54]. A study among young Black sexual minority men revealed that homonegativity may hinder obtaining PrEP prescriptions by negatively impacting communication with healthcare providers, contributing to medical mistrust, and leading to detachment and disbelief about PrEP [55].

Although there is extensive scientific evidence on barriers to PrEP use, the role of gay community connectedness remains under-researched in PrEP research. Moreover, there is a potential mediating role of attitudes toward PrEP and internalized homonegativity in the relationship between gay community connectedness and PrEP use, which is yet to be studied. This study aims to address this gap by determining the potential mediational role of attitudes towards PrEP and internalized homonegativity between gay community connectedness and PrEP use among MSM living in Georgia. We hypothesize that positive attitudes toward PrEP and reduced levels of internalized homonegativity could enhance PrEP use while acting as mediators in the relationship between gay community connectedness and PrEP use. Identifying salient pathways could support developing interventions to enhance PrEP use among MSM in Georgia.

## Materials and Methods

### Data Source

Data were obtained from an online cross-sectional survey conducted among 239 MSM in Georgia, a southern U.S. state, in June 2020. Among the total 239 participants, 173 participants were HIV negative, and PrEP use data were available for 121 participants. We included 121 participants in the data analysis for this study. Fifty-two (52) participants were excluded from our final analysis because they didn’t answer the question about PrEP use during their last sexual encounter. A sensitivity analysis was conducted to identify differences between excluded and included participants based on their sociodemographic characteristics, and it was found that there were no statistically significant differences (Supplementary Table 1).

### Participants and Data Collection Procedure

The study participants were recruited using three geosocial networking apps (GSNA) commonly used by MSM in Georgia (Grindr, Jack’d, Scruff) and were selected based on specific criteria. The criteria included identifying as MSM (i.e., gay, bisexual, pansexual, or generally as a man who has sex with men), being assigned male sex at birth, being 18 years or older, and being proficient in reading and writing English. The sampling frame was developed by detecting the frequently used GSNAs among MSM in Georgia, followed by finalization of the preliminary GSNA list. Consequently, the final selection of three GSNAs (i.e., Grindr, Jack’d, Scruff) was done based on the largest user concentration as described by Gibbs et al. [56].

To recruit the study participants, advertisements were broadcast on the three selected GSNAs to reach the maximum number of participants within the study’s geographic sampling frame. These advertisements guided the potential participants to screen for eligibility through an online eligibility screener developed for this study. A unique study identification (ID) code was generated for each screened participant, and contact information was collected for participation in the study. The study contact information manager (CIM) managed the information. He also sent a list of new eligible unique study IDs to the survey data manager (SDM), who then generated a survey link for each unique ID and sent it to CIM for distribution to the participants. The CIM then shared the survey link with the participants, along with directions for use, through which the participants entered the survey website and completed the survey following informed consent. Each participant received a $25 downloadable gift card as an incentive for participating in the study. Responses of the participants were stored in Qualtrics. The study received approval from the Institutional Review Board of the University of Georgia.

### Description of the Variables

Attitudes Towards PrEP We measured the participants’ attitudes toward PrEP using a “3-item Attitude Towards Pre-Exposure Prophylaxis (PrEP) scale” (Cronbach’s alpha = 0.739). The three items of the scale were “Gay and bisexual men should take PrEP,” “PrEP is likely to work,” and “PrEP will not probably have serious side effects.”

Gay Community Connectedness The study utilized the “8-item Identification and Involvement with the Gay Community scale” (Cronbach’s alpha = 0.795) to evaluate the association with and perceived connection to the gay community among MSM. The scale consists of eight self-report items. Items 1–4 indicate their association with the gay community, and items 5–6 and 7 relating to the frequency of accessing LGBTQ related media content (including paper, magazine, through both traditional and digital formats), and attending LGBTQ organizational activities, such as meetings, fund-raisers, political activities, and visiting gay bars. Item 8 assesses the number of gay or bisexual friends in their social network [57].

*Internalized homonegativity scale*: The study used nine items “Internalized Homophobia Scale (Cronbach’s alpha = 0.894)” [58], with responses on a 5-point scale (1 = Strongly Disagree to 5 = Strongly Agree). For example, sample items are: ‘I wish I weren’t gay/bisexual/pansexual’, ‘I have tried to stop being attracted to men in general.’ Possible scores ranged from 9 to 45. A higher score indicated greater internalized homonegativity.

### Outcome Measures

Pre-exposure Prophylaxis (PrEP) Use The outcome variable, pre-exposure prophylaxis (PrEP) use, as defined by the question “the last time you had sex, were you taking PrEP, like Truvada?” (Yes vs. No).

### Covariates

Covariates included participants’ age, race, education level, employment status, health insurance status, and geographical location. All the variables were self-reported. Some variables were recoded before analyzing the data. For instance, we combined education into four groups: “≤ high school”, “some college”, “Bachelor’s”, and “Higher” to simplify the analysis and manage small sample sizes. Similarly, we divided age into four groups (18–25, 26–35, 36–50, ≥ 50) based on our findings in the sample. The covariates were selected based on the literature review that were related to PrEP use to reduce the confounding effect in our results and better understand the effects of the exposure variables we’re focusing on [59, 60].

### Statistical Analysis

Chi-Square test was done to investigate the association between socio-demographic variables and “PrEP use among MSM in GA”. We conducted a sensitivity analysis between the included and excluded HIV-negative Men who have Sex with Men. We also conducted path analysis to determine the association between gay community connectedness (GCC) (exposure), attitudes towards PrEP and internalized homonegativity (mediator), and PrEP use (outcome), both crude and adjusted for covariates (sociodemographic variables). We obtained direct and indirect associations between gay community connectedness, internalized homonegativity, attitudes towards PrEP, and PrEP use. We reported both crude and adjusted beta (β) coefficients. P-value < 0.05 was considered statistically significant for all analyses. All analyses were done utilizing Stata 17.0.

## Results

Table 1 represents the distribution of sociodemographic variables of the participants (*N* = 121) and by PrEP use status. PrEP use was statistically different by participants’ age (*p* = 0.044), with higher PrEP use observed among the 36–50 (32.7%) and 26–35 (30.6%) age groups compared to other age groups. PrEP use was also statistically different by the race of the participants (*p* < 0.001), with White participants constituting the highest proportion (77.6%) among those who use PrEP at last sex. Participants with health insurance (87.8%) tended to use PrEP more compared to those without insurance (*p* = 0.002).

**Table 1:** Sociodemographic variables and PrEP use among HIV-negative men who have sex with men in Georgia, (N = 121)

Characteristics	Overall N = 121	PrEP Use		p-value
		No n = 72	Yes n = 49	
Gay community connectedness, mean (SD)	21.2 (5.3)	20.3 (5.3)	22.4 (5.2)	** 0.037**
Internalized Homonegativity, mean (SD)	17.1 (7.8)	19.1 (8.2)	14.3 (6.2)	**< 0.001**
Attitude towards PrEP, mean (SD)	11.7 (2.1)	10.8 (2.04)	13.0 (1.4)	**< 0.001**
Age (Years)	n (%)	n (%)	n (%)	** 0.044**
18–25	29 (24.0%)	20 (27.8%)	9 (18.4%)	
26–35	48 (39.7%)	33 (45.8%)	15 (30.6%)	
36–50	30 (24.8%)	14 (19.4%)	16 (32.7%)	
≥ 50	14 (11.6%)	5 (6.9%)	9 (18.4%)	
Race				**< 0.001**
African American	34 (28.1%)	30 (41.7%)	4 (8.2%)	
White	70 (57.9%)	32 (44.4%)	38 (77.6%)	
Other	17 (14.0%)	10 (13.9%)	7 (14.3%)	
Education				0.57
≤High School	14 (11.6%)	9 (12.5%)	5 (10.2%)	
Some College	35 (28.9%)	20 (27.8%)	15 (30.6%)	
Bachelor	44 (36.4%)	29 (40.3%)	15 (30.6%)	
Higher	28 (23.1%)	14 (19.4%)	14 (28.6%)	
Employment status				0.53
Unemployed	28 (23.1%)	17 (23.6%)	11 (22.4%)	
Full-time	73 (60.3%)	41 (56.9%)	32 (65.3%)	
Part-time	20 (16.5%)	14 (19.4%)	6 (12.2%)	
Health Insurance				**0.002**
No	33 (27.3%)	27 (37.5%)	6 (12.2%)	
Yes	88 (72.7%)	45 (62.5%)	43 (87.8%)	
Location				0.56
Urban	85 (70.2%)	52 (72.2%)	33 (67.3%)	
Rural	36 (29.8%)	20 (27.8%)	16 (32.7%)	

*Bolded p-values are statistically significant at p<0.05

Table 2 presents direct associations between gay community connectedness (GCC), internalized homonegativity, attitudes towards PrEP, and PrEP use. After adjusting for sociodemographic characteristics (i.e., age, race, education, employment status, health insurance, and location), attitude towards PrEP was positively associated with PrEP use (β = 0.11; *p* < 0.001), and gay community connectedness was also positively associated with PrEP attitudes (β = 0.17, *p* < 0.001). On the other hand, gay community connectedness was negatively associated with internalized homonegativity (β = −0.71, *p* < 0.001), and internalized homonegativity was negatively associated with PrEP use (β = −0.01, *p* = 0.031). However, gay community connectedness was not significantly associated with PrEP use (β = −0.01; *p* = 0.196).

**Table 2:** Direct association between gay community connectedness (GCC), internalized homonegativity, attitude towards prep, and PrEP use among HIV-negative men who have sex with men in georgia, (*N* = 121)

	Crude β	*p*-Value	Adjusted β	*p*-Value
GCC ◊ PrEP Use	0.01	0.214	−0.01	0.196
GCC ◊Attitude Towards PrEP	**0.16**	**< 0.001**	**0.17**	**< 0.001**
GCC ◊Internalized Homonegativity	**−0.70**	**< 0.001**	**−0.71**	**< 0.001**
Internalized Homonegativity ◊PrEP Use	**−0.01**	**0.015**	**−0.01**	**0.031**
Attitude towards PrEP ◊ PrEP Use	**0.12**	**< 0.001**	**0.11**	**< 0.001**

*Adjusted for age, race, education, employment status, health insurance status, and rural/urban location

**Bolded estimates are statistically significant at *p* < 0.05

**Fig. 1:**
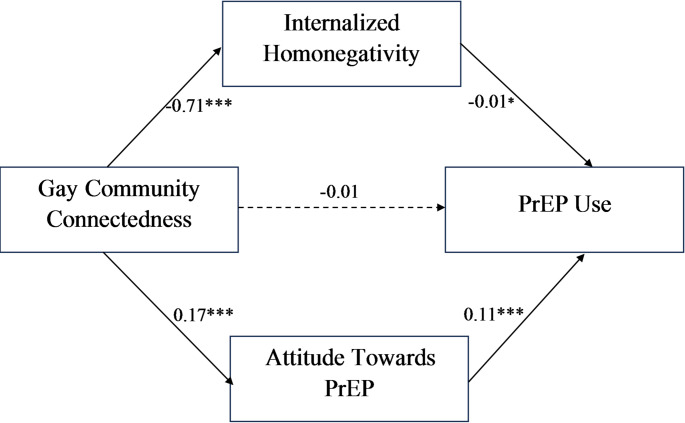
Mediating Pathway between Gay Community Connectedness (GCC), Attitude Towards PrEP, and PrEP use among men who have sex with men. ***p < 0.001, **p < 0.01, *p < 0.05. Solid arrows (→) indicate statistical significance at p<0.05

Table 3 shows the indirect association between gay community connectedness, internalized homonegativity, Attitude towards PrEP, and PrEP Use. The finding revealed that the indirect effects of gay community connectedness on PrEP use through PrEP attitudes (β = 0.01, *p* < 0.001) and internalized homonegativity (β = 0.02, *p* = 0.041) were statistically significant (Fig. 1).

**Table 3:** Indirect association between gay community connectedness (GCC), internalized homonegativity, attitude towards prep, and PrEP use among HIV-negative men who have sex with men in georgia, (*N* = 121)

Mediator	Crude β	*p*-Value	Adjusted β	*p*-Value
Internalized Homonegativity	**0.01**	**0.024**	**0.02**	**0.041**
Attitude towards PrEP	**0.02**	**< 0.001**	**0.01**	**< 0.001**

*Adjusted for age, race, education, employment status, health insurance status, and rural/urban location

**Bolded estimates are statistically significant at *p* < 0.05

## Discussion

The findings of this study revealed that the relationship between gay community connectedness and PrEP use among MSM in Georgia is mediated by attitudes towards PrEP and internalized homonegativity. Gay community connectedness was found to be significantly associated with PrEP attitudes (positive) and internalized homonegativity (negative). Statistically significant associations were also observed between attitudes towards PrEP and PrEP use (positive), and internalized homonegativity and PrEP use (negative). Though the direct association of gay community connectedness with PrEP use was not statistically significant, the indirect effect was found to be statistically significant. The findings suggest that gay community connectedness assists in shaping an individual’s attitudes toward PrEP and reducing internalized homonegativity, which influences PrEP use, demonstrating a mediating role of PrEP attitudes and internalized homonegativity in this relationship. Moreover, this study focused on MSM in Southern U.S. states like Georgia, where conventional values and negative attitudes towards LGBTQ + people more often influence community connectedness. Previous research has already explored the relationship between attitudes toward PrEP and internalized homonegativity on PrEP use [59, 61, 62]. This study extends existing research by applying a mediation framework to examine how community connectedness and stigma influence PrEP attitudes and uptake among MSM in Georgia, a region marked by high HIV burden and structural stigma. The findings of this study, grounded in a specific geographic and cultural context, provide valuable insights into the role of community connectedness and stigma on PrEP attitudes and uptake.

In our study, we observed a significant association between gay community connectedness and attitudes toward PrEP, which supports the idea that community or social networks play a core role in shaping health behaviors. For example, a U.S. study reported that 27.4% of the study participants heard about PrEP through social media, and 26.8% from friends, indicating that social connections are common sources of PrEP information and change agents for PrEP attitudes. Therefore, social or community connections can play an important role in publicizing and raising awareness of PrEP [59], reinforcing PrEP interest and intent, enhancing insights into PrEP benefits, reducing PrEP stigma, and building supportive community norms concerning PrEP acceptance among key populations at risk for HIV [63]. Consequently, social network interventions have shown encouraging outcomes in improving the uptake of biomedical approaches to HIV prevention, including PrEP [64]. However, the prevailing stigma within social networks could negatively impact PrEP attitudes and willingness to use [51] as well as contribute to the diffusion of negative experiences of discrimination and concerns about PrEP within the network, creating medical mistrust [65–67]. Our study also found a significant positive association between PrEP attitudes and PrEP use, indicating that positive attitudes towards PrEP (i.e., believing PrEP is effective and has no serious side effects) lead to a higher likelihood of PrEP use and adherence. One US-based study demonstrated an association between PrEP perception or attitude and willingness to use PrEP [68]. Furthermore, positive attitudes towards PrEP were found to be associated with increased PrEP awareness that leads to positive health beliefs and health behavior, including the intention of PrEP uptake [69]. Therefore, our findings suggest that improving gay community connectedness and increasing positive PrEP attitudes could contribute to PrEP uptake and persistence among MSM.

A significant negative association was identified between gay community connectedness and internalized homonegativity in our study, indicating the protective function of community connectedness against internalized homonegativity. Prior research has demonstrated the likelihood of lowering internalized homonegativity through connection with the gay community [30, 45, 70]. Therefore, strong community connection leads to the enhancement of a sense of inclusion in society, societal acceptance and validation of identity, facilitates access to social support, and improves self-efficacy and self-esteem, thus buffering against internalized homonegativity [71, 72]. Similarly, internalized homonegativity is also negatively associated with PrEP use [73]. Taken together, these findings indicate that an increase in homonegativity decreases PrEP awareness, creates barriers to health care access (including access to PrEP), increases PrEP stigma, and thus reduces the likelihood of PrEP use [74–76].

This study found no statistically significant direct relationship between gay community connectedness and PrEP use (β = −0.01; *p* = 0.196). Nevertheless, this does not undermine the theoretical implications of the model. The mediating pathways, particularly attitudes toward PrEP and internalized homonegativity, showed significant relationships with PrEP use. This indicates that gay community connectedness may indirectly affect PrEP use by shaping social norms, lowering stigma, or modifying health beliefs. While strong connections within the community could advance supportive norms that promote PrEP use, in others, they might boost stigma or spread misinformation that could hinder PrEP uptake. Therefore, gay community connectedness exhibits valuable opportunities for implementing interventions, even if it doesn’t directly predict behavior in all contexts. Programs aimed at advancing positive community norms, promoting peer education, and reducing stigma related to PrEP use can be effectively employed in these settings. Subsequently, while our findings emphasize the importance of a deeper understanding of community connectedness, they also acknowledge the model’s role in guiding public health strategies to enhance PrEP engagement.

This study also highlighted inequalities in PrEP use across the sociodemographic variables. White participants were found to be significantly more likely to use PrEP than African Americans and other racial groups. A similar pattern was observed among U.S. MSM, with 68% of White MSM reporting PrEP use, which is higher compared to 55% among Black MSM [77]. Moreover, PrEP use was significantly higher among individuals with health insurance than among those without insurance. A U.S. PrEP Demonstration Project reported barriers to PrEP access among non-white, uninsured participants [78]. These racial and socio-economic inequalities in PrEP use could exacerbate the disparities in the HIV epidemic [79].

This study identifies important factors and their patterns of relationships in influencing PrEP use. The findings provide crucial guidance for formulating a targeted PrEP intervention that incorporates network-based approaches and addresses the factors shaping attitudes toward PrEP, as well as reducing internalized homonegativity. Social network-based interventions, including peer-led approaches and online communities (e.g., Facebook networks), can enhance PrEP knowledge and awareness, reduce stigma, dispel misconceptions and concerns about PrEP, build confidence in PrEP, and promote preventive behaviors [25, 80, 81]. Consequently, all these factors promote a positive attitude towards PrEP and lower the internalized stigma and homonegativity, which in turn increases PrEP use among MSM. A study (PrEP Chicago) conducted among young Black MSM in Chicago showed the effectiveness of social networks in the adoption of HIV prevention strategies, including PrEP [82]. Additionally, the intervention should address structural barriers to PrEP use, such as racial and socio-economic disparities, by expanding healthcare coverage and reducing the cost of PrEP medicine to an affordable price.

### Limitations

The study has some limitations that need to be addressed through future research. First, the study followed a cross-sectional approach, hence, it is not possible to suggest causality or establish temporality between gay community connectedness, attitudes towards PrEP, and PrEP use. A longitudinal study could determine the causality and temporal relationships between the variables. Second, the study included self-reported data (PrEP use), so it is subject to recall bias or social desirability bias. Third, all study participants were recruited through GSNs and live in Georgia. Therefore, some participants who do not use the selected GSNs may be excluded, which could limit the generalizability of the findings to MSM outside of Georgia. This study did not assess the potentially negative aspects of gay community connectedness, such as misinformation, stigma, medical mistrust, etc. Therefore, future studies could investigate both supportive and hindering dynamics within community networks, particularly in the current U.S. socio-political context (e.g., politicization of health, anti-LGBTQ + policies). Furthermore, one item in the PrEP attitudes scale says, “Gay and bisexual men should take PrEP.” This might mix up an individual’s sexual identity with their risk of getting HIV, which is better looked at through their sexual behaviors. For example, some gay or bisexual people might not be at high risk if they are in monogamous relationships, identify as asexual, or are not the one who takes on the active role in sex. Future research should examine attitudes towards PrEP based on individuals’ behaviors, rather than just their identity. Another limitation of this study is that it only asked participants one question about using PrEP during their last sexual encounter. This only demonstrates their recent behavior, but it doesn’t tell us about their overall sexual activity or if they are eligible for PrEP. The study didn’t collect details about their sexual risk behaviors, like the number of sexual partners, the type and frequency of sex, condom use, or STI history. This information is essential for determining who needs PrEP according to CDC guidelines [83]. Future studies should gather more information about HIV risk to evaluate PrEP use better.

## Conclusions

To our knowledge, very little research has been conducted on gay community connectedness, internalized homonegativity, PrEP attitudes, and PrEP use, particularly among MSM in Georgia. This study highlights the mediating role of attitudes towards PrEP and internalized homonegativity between gay community connectedness and PrEP use among MSM. The findings indicate an indirect influence of gay community connectedness on PrEP use through the mediators “attitudes towards PrEP” and “internalized homonegativity”, highlighting the role of positive attitudes towards PrEP and reduced internalized homonegativity in enhancing PrEP use among MSM. Strong gay community connectedness leads to positive attitudes toward PrEP, which might be due to better access to PrEP information and the influence of peers’ motivation for positive health behavior. Consequently, a positive attitude strongly influences PrEP use through a supportive community environment. Additionally, community connectedness reduces PrEP stigma through increasing coping skills, PrEP knowledge, and mental health support, thus subsequently enhancing their willingness and intention to use PrEP. The findings highlight the importance of community networks and belongingness to support the development of positive attitudes towards PrEP and reduce internal homonegativity among MSM to enhance PrEP use and reduce HIV disparities in a high HIV burden region like Georgia. Future studies should explore the other mediators between gay community connectedness and PrEP use, such as stigma and PrEP awareness. Moreover, the findings could guide the development of interventions for MSM to improve PrEP use.

## Supplementary Information

Below is the link to the electronic supplementary material.

Supplementary Material 1

## Data Availability

Data can be obtained from Dr. Jeremy J. Gibbs (jeremy.gibbs@uga.edu) by reasonable request.

## Code Availability

 Codes can be obtained from Dr. Mohammad Rifat Haider (haider@uga.edu) by reasonable request.
