# Supplementary material for: Gay Community Connectedness, Internalized Homonegativity, and HIV Pre-Exposure Prophylaxis (PrEP) Attitudes and Use Among Men Who Have Sex with Men in Georgia: A Mediation Analysis
Source: AIDS Behav. 2025 Sep 12;30(2):319–28. doi: 10.1007/s10461-025-04870-0 (PMC12929244; doi:10.1007/s10461-025-04870-0)
Supplement: Supplementary file 1 — Supplementary Material 1 [file 10461_2025_4870_MOESM1_ESM.docx]

**Supplementary Materials**

**Supplementary Table 1.** **Sensitivity Analysis between the included and excluded HIV-negative Men who have Sex with Men, (N=173)**

| **Characteristics** | **Excluded**  52 (30.1%) | **Included**  121 (69.9%) | **Total**  173 (100.0%) | **p-value** |
| --- | --- | --- | --- | --- |
| **Gay community connectedness**, mean (SD) | 21.0 (5.1) | 21.2 (5.3) | 21.1 (5.3) | 0.833 |
| **Internalized Homonegativity**, mean (SD) | 19.0 (8.5) | 17.1 (7.8) | 17.7 (8.0) | 0.162 |
| **Age (Years)** | **n (%)** | **n (%)** | **n (%)** | 0.080 |
| 18-25 | 15 (28.8%) | 29 (24.0%) | 44 (25.4%) |  |
| 26-35 | 26 (50.0%) | 48 (39.7%) | 74 (42.8%) |  |
| 36-50 | 4 (7.7%) | 30 (24.8%) | 34 (19.7%) |  |
| ≥50 | 7 (13.5%) | 14 (11.6%) | 21 (12.1%) |  |
| **Race** |  |  |  | 0.116 |
| African American | 23 (44.2%) | 34 (28.1%) | 57 (32.9%) |  |
| White | 23 (44.2%) | 70 (57.9%) | 93 (53.8%) |  |
| Other | 6 (11.5%) | 17 (14.0%) | 23 (13.3%) |  |
| **Education** |  |  |  | 0.157 |
| ≤High School | 8 (15.4%) | 14 (11.6%) | 22 (12.7%) |  |
| Some College | 22 (42.3%) | 35 (28.9%) | 57 (32.9%) |  |
| Bachelor | 16 (30.8%) | 44 (36.4%) | 60 (34.7%) |  |
| Higher | 6 (11.5%) | 28 (23.1%) | 34 (19.7%) |  |
| **Employment status** |  |  |  | 0.878 |
| Unemployed | 35 (71.4%) | 85 (70.2%) | 120 (70.6%) |  |
| Full-time | 14 (28.6%) | 36 (29.8%) | 50 (29.4%) |  |
| Part-time |  |  |  | 0.992 |
| **Health Insurance** | 12 (23.1%) | 28 (23.1%) | 40 (23.1%) |  |
| No | 31 (59.6%) | 73 (60.3%) | 104 (60.1%) |  |
| Yes | 9 (17.3%) | 20 (16.5%) | 29 (16.8%) |  |
| **Location** |  |  |  | 0.962 |
| Urban | 14 (26.9%) | 33 (27.3%) | 47 (27.2%) |  |
| Rural | 38 (73.1%) | 88 (72.7%) | 126 (72.8%) |  |
